# Identification of Biomarkers Associated with Heart Failure Caused by Idiopathic Dilated Cardiomyopathy Using WGCNA and Machine Learning Algorithms

**DOI:** 10.1155/2023/2250772

**Published:** 2023-04-25

**Authors:** Mengyi Sun, Linping Li

**Affiliations:** ^1^Department of Clinical Laboratory, Jining First People′s Hospital, Jining, Shandong, China; ^2^Institute of Cardiovascular Diseases of Jining Medical Research Academy, Jining First People′s Hospital, Jining, Shandong, China

## Abstract

**Background:**

The genetic factors and pathogenesis of idiopathic dilated cardiomyopathy-induced heart failure (IDCM-HF) have not been understood thoroughly; there is a lack of specific diagnostic markers and treatment methods for the disease. Hence, we aimed to identify the mechanisms of action at the molecular level and potential molecular markers for this disease.

**Methods:**

Gene expression profiles of IDCM-HF and non-heart failure (NF) specimens were acquired from the database of Gene Expression Omnibus (GEO). We then identified the differentially expressed genes (DEGs) and analyzed their functions and related pathways by using “Metascape”. Weighted gene co-expression network analysis (WGCNA) was utilized to search for key module genes. Candidate genes were identified by intersecting the key module genes identified via WGCNA with DEGs and further screened via the support vector machine-recursive feature elimination (SVM-RFE) method and the least absolute shrinkage and selection operator (LASSO) algorithm. At last, the biomarkers were validated and evaluated the diagnostic efficacy by the area under curve (AUC) value and further confirmed the differential expression in the IDCM-HF and NF groups using an external database.

**Results:**

We detected 490 genes exhibiting differential expression between IDCM-HF and NF specimens from the GSE57338 dataset, with most of them being concentrated in the extracellular matrix (ECM) of cells related to biological processes and pathways. After screening, 13 candidate genes were identified. Aquaporin 3 (AQP3) and cytochrome P450 2J2 (CYP2J2) showed high diagnostic efficacy in the GSE57338 and GSE6406 datasets, respectively. In comparison to the NF group, AQP3 was significantly down-regulated in the IDCM-HF group, while CYP2J2 was significantly up-regulated.

**Conclusion:**

As far as we know, this is the first study that combines WGCNA and machine learning algorithms to screen for potential biomarkers of IDCM-HF. Our findings suggest that AQP3 and CYP2J2 could be used as novel diagnostic markers and treatment targets of IDCM-HF.

## 1. Introduction

As a non-ischemic heart disease, dilated cardiomyopathy (DCM) is characterized by abnormal myocardial structure and function [1, 2]. It is the most common indication of heart transplantation and the third-most prevalent pathogenic factor responsible for heart failure (HF) [3, 4]. Idiopathic and familial diseases are the most common causes of DCM. According to a previous report, around 70% DCM cases are idiopathic [4]. Idiopathic dilated cardiomyopathy (IDCM) is a myocardial disease of unknown etiology, which can only be diagnosed if a secondary cause is excluded. Among HF patients, IDCM patients are younger and more at risk than cohorts with other etiologies (ischemic, hypertensive, and valvular) [5, 6]. The pathogenesis and genetic factors involved in the development of IDCM-induced HF (IDCM-HF) are not fully understood, and there is a lack of specific diagnostic markers and hardly any specific treatments for IDCM-HF. Therefore, it is crucial to unveil the specific molecular mechanism of IDCM-HF and to promote the diagnosis and precision treatment of the disease [7].

In recent years, gene sequencing and bioinformatics methods have resulted in new ideas for the elucidation of the mechanisms of disease development, disease diagnosis, and personalized precision medicine development. Wang et al. validated the diagnostic value of *MYG1*, *FLOT1*, and *ATG13* and proposed potential immunological mechanisms, biomarkers, and treatment targets in DCM patients with HF [8]. Qiu et al. investigated the mechanism of action of long noncoding RNAs (lncRNAs) including LING01-AS1, AC061961.2, and RP11-13e1.5, which were implicated with the development of DCM with HF [9]. Zhang et al. found that ASPN, CD163, IL10, and LUM could predict the occurrence of DCM [10]. Despite this, there are few studies on IDCM-HF. Huang et al. [11] explored the key genes associated with the development of IDCM-HF, but their conclusions need to be examined further as the results have not been verified.

In this study, we combined weighted gene co-expression network analysis (WGCNA) with machine learning algorithms, including support vector machine-recursive feature elimination (SVM-RFE) and least absolute shrinkage and selection operator (LASSO), to screen hub genes of IDCM-HF samples for the first time. Results were then verified using the external dataset. Target genes that could be reliably used for exploring the pathogenesis, diagnosing, and managing IDCM-HF.

## 2. Materials and Methods

### 2.1. Data Source

**Figure 1:**
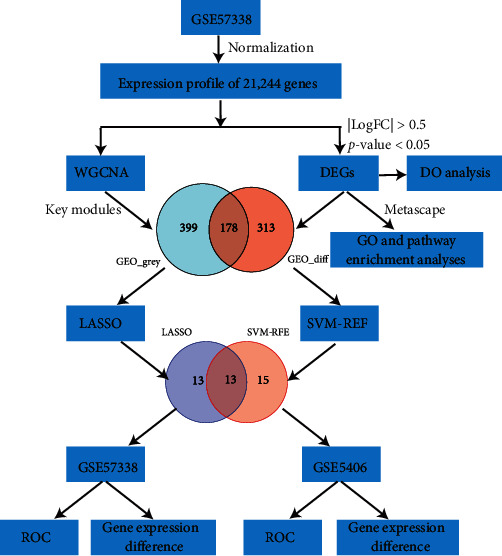
Flow chart of the study.

Figure 1 illustrates the workflow chart of data preparation, processing, analysis, and validation. Two datasets (GSE57338 [12] and GSE5406 [13]) were acquired from the Gene Expression Omnibus (GEO) database (http://www.ncbi.nlm.nih.gov/geo/). Both of the datasets were novel myocardial gene expression signatures of HF caused by IDCM. The GSE57338 dataset (GPL11532 platform), which was updated in 2018, was used for expression profiling analyses of the heart left ventricle of 82 IDCM-HF patients and 136 non-heart failure (NF) controls. The GSE5406 dataset (GPL96 platform) found data regarding expression profiling analyses of the heart left ventricle of 86 IDCM-HF patients and 16 NF controls. The basic sample information from the datasets was shown in Table 1. There were only two datasets (GSE57338 and GSE5406) on HF induced by IDCM found in the GEO database, so we selected the GSE57338 dataset with a larger sample size as the training group and GSE5406 as the verification group, in place of the combined analysis to validate the results.

### 2.2. Identification and Analysis of DEGs

Firstly, we adjusted the threshold with |log2 fold change (FC)| > 1 and |log2 FC| > 0.5, respectively, to select the cut-off point. Then we utilized the LIMMA package of R to identify the DEGs between ICDM-HF and NF samples in the GSE57338 dataset. Disease Ontology (DO) enrichment analysis was performed using the DOSE package of R. The functions and related pathways of DEGs were analyzed by “Metascape” [14]. During functional analysis, we included gene ontology (GO) analysis; it included an analysis of the molecular functions (MF), cellular components (CC), and biological processes (BP). During pathway analysis, we also performed Kyoto Encyclopedia of Genes and Genomes (KEGG), canonical pathway, Reactome pathway, Wiki pathway, and PANTHER pathway analyses.

### 2.3. WGCNA Analysis

First, we constructed a clustering tree for samples to identify and remove outliers. Afterwards, the gene expression data of the GSE57338 dataset were analyzed via WGCNA [15]. We performed “pickSoftThreshold” in WGCNA to compute *β* values (range: 1–20) and select the optimal soft threshold. For network construction, the topological overlap matrix was created via transformation of the adjacency matrix, and the gene tree and color of modules were established based on the dissimilarity degree. Dynamic tree cutting was implemented for division of the generated modules, after which comparable modules were combined. Key modules were picked according to the correlation between the module members and the significance of genes. Genes from key modules were used for subsequent analysis.

### 2.4. Identification of Hub Genes in IDCM-HF

The intersecting genes between DEGs and genes in the key modules via WGCNA were identified to obtain candidate genes. Then, we implemented LASSO and SVM-RFE by adopting the GLMnet [16] and e1071 [17] packages in R, respectively, to further identify hub genes, which were the genes identified by both LASSO and SVM-RFE.

### 2.5. Hub Genes Verification

Hub genes were verified by evaluating the diagnostic efficacy using receiver operating characteristic (ROC) curves and appraising the expression profile in the training group (GSE57338) and the verification group (GSE5406). Firstly, we plotted ROC curves using pROC packages [18] and determined the area under ROC curves (AUC). An AUC value more than 0.8 signified that the data was a good fit for the gene, and a *P*-value less than 0.05 indicated that the value was of statistical significance. Secondly, we confirmed the difference in hub gene expression in the IDCM-HF and NF groups. The validated genes were identified as robust diagnostic biomarkers for IDCM-HF.

## 3. Results

### 3.1. Identification of DEGs

**Figure 2:**
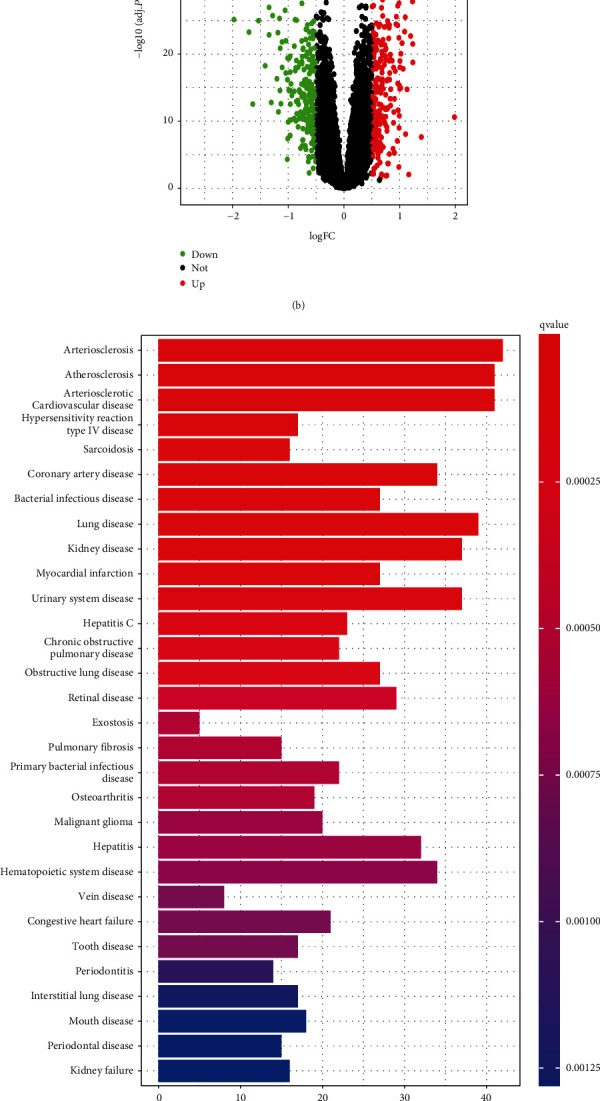
Correlation analysis of DEGs in the NF and IDCM-HF groups in the GSE57338 dataset. (a) Heatmap of the 50 most significantly up- and down-regulated DEGs. (b) Volcanic map. (c) Histogram of the top 30 diseases observed during DO analysis. (d) Bubble charts of the top 30 diseases observed during DO analysis. NF: non-heart failure; IDCM-HF: idiopathic dilated cardiomyopathy-induced heart failure; GO: gene ontology.

The GSE57338 dataset downloaded from GEO contains 20,134 genes in 218 left ventricular myocardia from 82 IDCM-HF patients and 136 NF controls. The DEGs were identified between ICDM-HF and NF samples in the GSE57338 dataset with selected thresholds of |log2 FC| > 0.5 and adjusted *p*-value <0.05. There were totally 490 DEGs identified based on the criteria mentioned in the Materials and Methods section. The heat map shows the expression profile of the top 50 differential genes (Figure 2(a)). 269 and 221 genes of DEGs exhibited significantly higher and lower expression, respectively, in the IDCM-HF patients compared with the controls, as displayed by the volcano map (Figure 2(b)).

### 3.2. DO Enrichment Analysis

We performed DO enrichment analysis of DEGs and found that their expression was implicated with the occurrence of heart diseases such as atherosclerosis, atherosclerotic heart disease, myocardial infarction, coronary heart disease, and congestive HF (Figures 2(c) and 2(d)).

### 3.3. Functional Enrichment Analyses

**Figure 3:**
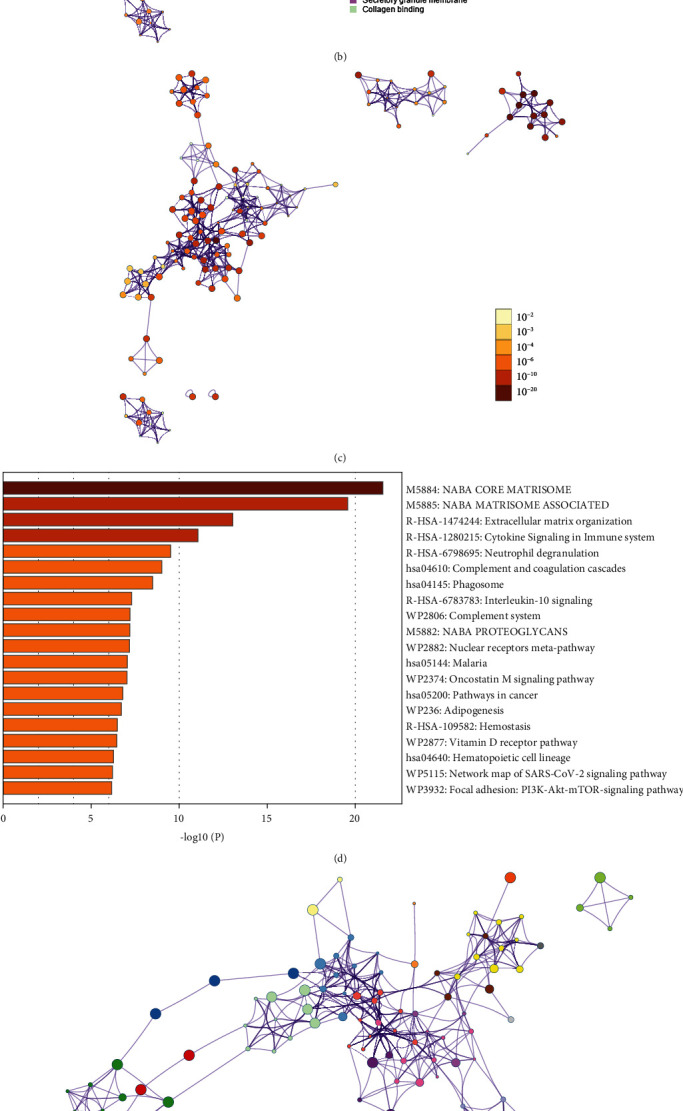
Functional enrichment analyses. (a) GO annotation. Construction of the GO functional annotation network based on the cluster ID (b) and *P*-value (c). (d) Related pathway analysis. Construction of the enrichment analysis network in accordance with the cluster ID (e) and *P*-value (f). GO: gene ontology.

To further explore the DEGs' biofunctions, GO annotation and pathway analysis were performed. As shown in Figures 3(a), 3(b), and 3(c), according to GO annotation, most of the DEGs were associated with the extracellular matrix (ECM); they were also associated with processes such as inflammatory response generation, glycosaminoglycan binding, response generation to cytokine stimulation, response generation to toxic substances and bacteria, and regulation of certain processes. Consistent with GO enrichment analysis, the pathways with which DEGs were associated were mainly related to the ECM, NABA_MATRISOME_ASSOCIATED, NABA_CORE_MATRISOME, and ECM organization-related pathways (Figures 3(d), 3(e), and 3(f)).

### 3.4. Establishment of a Network Based on WGCNA and Identification of Hub Modules

**Figure 4:**
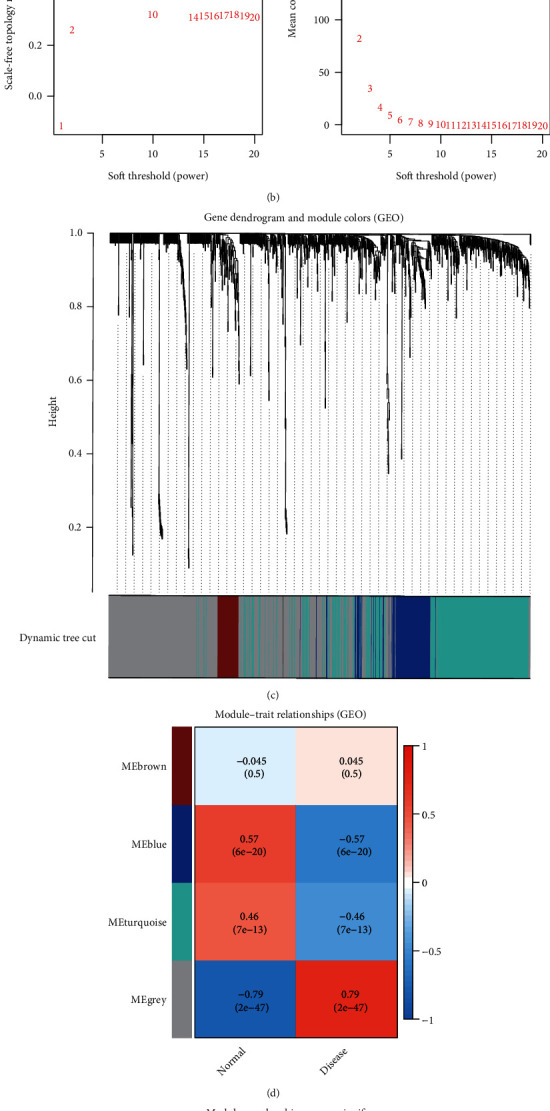
WGCNA of the GSE57338 dataset. (a) Clustering of samples for the detection of an outlier. (b) The left and right panels represent scale-free index and mean connectivity analyses, respectively, at different threshold powers. (c) Clustering dendrogram of genes and colors corresponding to each module. (d) Module–trait associations. Module membership vs. gene significance of the MEgrey module in the (e) NF and (f) IDCM-HF groups. NF: non-heart failure; IDCM-HF: idiopathic dilated cardiomyopathy-induced heart failure.

The detailed process of WGCNA analysis was shown in Additional file 3. After verifying the missing values, we constructed a sample clustering tree (Figure 4(a)) and selected 11 as the optimal soft threshold power. The *R*^2^ value was 0.896. Then, the network topology was analyzed with 1–20 threshold weights (Figure 4(b)). After merging similar modules, a total of four modules were identified (Figure 4(c)). Based on the module–trait associations (Figure 4(d)), the MEgrey module was picked as a key module. The scatter diagram (Figures 4(e) and 4(f)) shows the significance of the module membership versus gene in this module. In the NF and IDCM groups, the correlation coefficient was 0.86. The genes in MEgrey were subjected to further investigations.

### 3.5. Identification of Hub Genes

**Figure 5:**
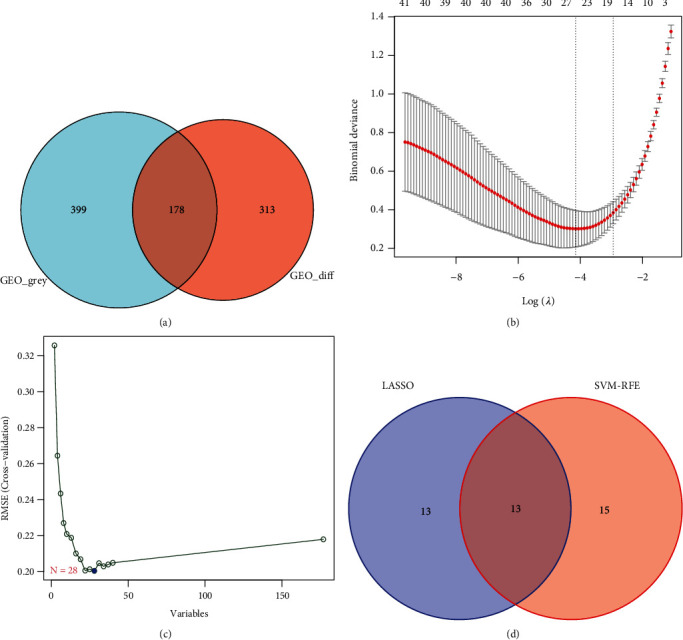
Identification of candidate genes. (a) Venn diagram showing the 178 intersecting genes between DEGs and genes in MEbrown. (b) LASSO algorithm for the screening of candidate feature genes. (c) SVM-RFE algorithm for the screening of candidate genes. (d) Venn diagram of 13 overlapping candidate genes among the genes identified by the SVM-RFE and LASSO algorithms. DEG: differentially expressed gene; SVM-RFE: support vector machine-recursive feature elimination; LASSO: least absolute shrinkage and selection operator.

We intersected the DEGs and genes from the MEgrey module identified using WGCNA and obtained 178 intersected genes (Figure 5(a)). We conducted LASSO and SVM-REF screening in order to further identify the hub genes. Through LASSO screening (Figure 5(b)), we obtained 26 genes (Table 1). Through SVM-REF screening (Figure 5(c)), we obtained 28 genes (Table 1). After the intersection of the genes screened using LASSO and SVM-REF, 13 hub genes were obtained (Figure 5(d), Table 1); these included *LCN6*, *SLC16A9*, *AQP3*, *C16orf89*, *USP13*, *EDNRB*, *CYP2J2*, *NT5E*, *CREB5*, *MPP3*, *MID1IP1*, *SLCO2A1*, and *DHCR24*.

### 3.6. Verification of Hub Genes

**Figure 6:**
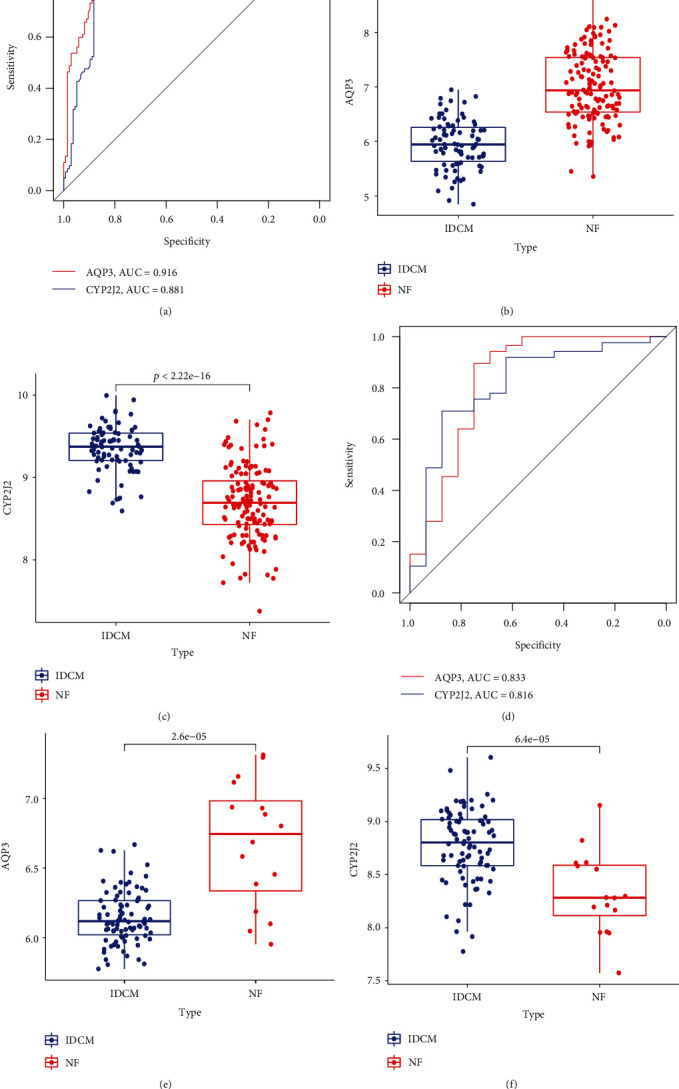
Verification of hub genes. (a) ROC curves of the hub genes in the GSE57338 dataset. Validation of expression levels of AQP3 (b) and CYP2J2 (c) in GSE53778. (d) ROC curves of the hub genes in GSE5406. Validation of expression levels of AQP3 (e) and CYP2J2 (f) in GSE5406. ROC: receiver operating characteristics.

We further evaluated the diagnostic efficacy of these 13 genes using ROC curves in the GSE57338 dataset. As exhibited by Figure 6(a) and Additional file 1, these genes were associated with high levels of accuracy. The AUC was greater than 0.8 for all genes except *DHCR24*, for which the AUC was 0.75. Meanwhile, we appraised the expression profile of the 13 genes. As shown in Figures 6(b) and 6(c), and Additional file 2, these genes displayed significantly differential expression between the NF and IDCM groups. In order to further appraise the diagnostic significance of candidate genes and obtain reliable diagnostic markers, we conducted validation with the external dataset GSE5406. In the GSE5406 dataset, two genes (AQP3 and CYP2J2) intersected with 13 target genes, and the results obtained for AQP3 and CYP2J2 were consistent with those obtained with GSE57338, and the corresponding AUC was 0.833 and 0.816, respectively (Figure 6(c)). In comparison with the NF group, AQP3 expression was down-regulated and CYP2J2 expression was up-regulated in the IDCM-HF group, and the differences were statistically significant (Figures 6(d), 6(e), and 6(f)).

## 4. Discussion

IDCM is the most prevalent cause of non-ischemic HF. In most cases, the mechanisms of action are unknown, and the main pathologic disorders involve patchy interstitial fibrosis, myocardial cell degeneration, and ventricular dilatation [19]. Myocardial injury, as well as genetic and environmental factors, might be the pathogenic mechanism of IDCM [20]. The heterogeneity of etiology of the disease makes it difficult to achieve a rapid diagnosis, but the use of high-throughput molecular biology techniques might represent a new solution. We used a combination of WGCNA, LASSO, and SVM-RFF for the first time, to screen the target genes associated with human IDCM-HF and performed external dataset validation. Our findings could shed new lights for diagnosing and managing IDCM-HF patients.

The authors of the GSE 57338 identified genes with distinct expression patterns between failing and non-failing hearts. The authors of the GSE5406 determined HF genes differentially expressed and found that a discrete set of cardiac transcription factors (TFs) was associated with human HF. In this bioinformatics analysis, based on the results of gene microasrray of GSE57338, we used WGCNA and machine learning algorithms to identify biomarkers associated with HF caused by IDCM and further verified the diagnostic efficacy and the expression profile of the hub genes in the dataset GSE 5406. The validated hub genes could shed new lights for diagnosing and managing IDCM-HF patients.

There were 490 DEGs in the IDCM-HF and NF control groups that were associated with atherosclerosis and atherosclerotic and coronary heart diseases. This is consistent with the findings of the following published studies. Recent studies suggest that the coronary atherosclerotic load was a significant factor predictive of major adverse cardiovascular events in non-ischemic DCM patients [21]. Studies show that the heart function in patients exhibiting a DCM-based deteriorating mechanism was likely to be associated with myocardial perfusion inadequacy and microvascular level of coronary artery disease [22], and the coronary microcirculation conditions for IDCM were consistent with the extent of progression of HF [23]; around 50% IDCM patients exhibited endothelial dysfunction of the coronary artery [24].

In accordance with the findings reported by Huang et al. [11], we found that most of the DEGs were associated with the extracellular matrix, which is a complicated network of fibrin (mainly types I and III collagen), elastic fiber, glycosaminoglycan, glycoprotein, and adhesive proteoglycan [25]. In case of a cardiac or extracardiac damage, ECM regulation might be crucial for the remodeling and fibrosis of ventricle. Gunja-Smith et al. were the first to demonstrate that an increase in the level of matrix deposition was caused by an increase in the collagen content in DCM. The newly produced collagen cannot form stable crosslinks, resulting in the dilatation of the ventricular wall [26]. In comparison to the healthy heart, the expression of metalloproteinases (MMPs) was up-regulated and that of its inhibitor was down-regulated in the heart of DCM patients. The up-regulation of MMPs could promote ECM degradation [27]. In addition, some studies have found that the increased level of serum tenascin C (TNC) in patients with DCM might aggravate cardiac fibrosis [27]. ECM functions both as a structural scaffold and as a center for the transmission of signals resulting in cascade reactions that are essential to the functioning of cells. In addition, as a repository of growth factors, it could release growth factors that regulate cellular behavior and activate the process of repair after injury, thereby playing an essential role in HF pathogenesis [28]. However, little is known about the role of specific ECM-dependent molecular pathways in the regulation of the ECM, and future studies need to examine this aspect.

AQPs constitute a highly conserved family of membrane channel proteins responsible for transmembrane water and uncharged small molecule transport. There have been reports regarding 13 human AQPs, from AQP0–AQp12, till date [29–33]. AQP3 is an aqua glycerol channel protein that can permeate through solutes such as water, glycerin, urea [34], transport H_2_O_2_ [35], and ammonia [36]. AQP3 is distributed extensively in the eye, inner ear, heart, lung, kidney, skin, gastrointestinal tract, reproductive tract, adipose tissues, and cartilage tissues [29, 37]. AQP3 is extensively involved in the development of many diseases. It was found to control water balance and urine concentration within the cells in the collecting duct. It also promotes cell migration, proliferation, and re-epithelialization during the healing of skin wounds [38–40]. The reduced expression of AQP3 during sepsis might play a role in immune cell migration [41]. It was also involved in the toll-like receptor 4-induced activation of macrophages during inflammation [30]. In addition, increased levels of AQP3 expression have been found in many cancers, and increased AQP3 expression enhances the proliferation, invasion, and migration of cancer cells and exacerbates the epithelial–mesenchymal transition-mediated cancer progression [42]. At present, there are few studies on the association between AQPs and cardiovascular disease. AQPs 1 and 3 are the most common endothelial AQPs. A loss of AQP1 is associated with endothelial dysfunction and atherosclerotic progression [43]. Increased AQP1 levels could lead to myocardial hypertrophy [44]. In human stomach cancer cells, up-regulation of AQP3 was associated with the increased expression of MMP2 and MMP9 [45]. AQP3 promotes ECM degradation [37]. The excessive degradation of MMPs could lead to an aneurysm or rupture in the left ventricle (LV), while excessive accumulation could lead to left ventricular stiffness, increased non-compliance, and HF [25]. In this work, we observed that AQP3 displayed lower expression in IDCM-HF patients. It is hypothesized that AQP3 might alleviate myocardial fibrosis and HF via the reduction in the MMP expression level and degradation of the ECM. This would provide a new direction to the research conducted in this field. It is crucial to better understand the activation, gating, and trafficking mechanisms of AQPs to facilitate the development of novel targeted therapies.

We also observed that CYP2J2 displayed a higher expression level in the IDCH-HF group than in the controls. Cytochrome P450 (CYP) is a monooxygenase located on the cell membrane, which can oxidize exogenous and endogenous substances in a pH-dependent manner. CYP2J2 is the only cyclooxygenase found in humans and is mainly expressed in the heart [46–49]. It converts arachidonic acid into epoxyeicosatrienoic acids (EETs), such as 5,6-, 8,9-, 11,12-, and 14,15-EETs. CYP2J2 and EETs have been repeatedly demonstrated to possess protective effects against various cardiovascular diseases. Previous reports have described the function of EETs during cardiac remodeling inhibition in cardiac hypertrophy, fibrosis, angiogenesis, inflammation, and apoptosis [49, 50]. The findings of another study indicated that overexpression of CYP2J2 promoted the production of EET, alleviated cardiac function impairment, and enhanced the fibrotic response in *CYP2J2* transgenic mice [51]. Ma et al. found that the overexpression of *CYP2J2* alleviated diabetic cardiomyopathy and tumor necrosis factor (TNF)-*α*-induced heart tissue damage [52]. CYP2J2 expression was up-regulated in the IDCH-HF group. This was thought to inhibit cardiac fibrosis and reduce cardiac remodeling. The mechanism of action of *CYP2J2* still needs to be explored further.

Several limitations are associated with this study. First, because specific cardiac tissues had to be used, the sample size was small. Second, only one microarray dataset was analyzed, which inevitably resulted in a deviation. Third, further in vitro and animal experiments need to be conducted to elaborate the mechanisms underlying the regulatory effects of AQP3 and CYP2J2 on IDCM-HF. Fourth, IDCM is a primary disease with hereditary, infectious, and autoimmune etiology, and its pathogenesis needs to be further explored according to the etiology. Fifth, the lack of clinical information in the datasets may lead to population bias.

## 5. Conclusion

In summary, we used WGCNA and the machine learning algorithm for the first time to identify biomarkers of IDCM-HF and obtained two hub genes, *AQP3* and *CYP2J2*, which have the potential to serve as targets for the diagnosis and management of IDCM-HF.

## Figures and Tables

**Table 1:** The basic sample information from GSE 57338 and GSE 5406.

GSE accession		GSE57338	GSE5406
NF			
	Mean age	49.36 ± 15.00	—
	Male	73	—
	Female	63	—
	Total patients	136	16
IDCM-HF			
	Mean age	51.16 ± 13.98	—
	Male	63	—
	Female	19	—
	Total patients	82	86

**Table 2:** The list of genes obtained using LASSO, SVM-REF, and the intersection of LASSO and SVM-REF.

No.	LASSO genes	SVM-REF genes	Inter genes
1	AQP3	AQP3	AQP3
2	C16orf89	C16orf89	C16orf89
3	CREB5	CREB5	CREB5
4	CYP2J2	CYP2J2	CYP2J2
5	DHCR24	DHCR24	DHCR24
6	DUSP13	DUSP13	DUSP13
7	EDNRB	EDNRB	EDNRB
8	MID1IP1	MID1IP1	MID1IP1
9	MPP3	MPP3	MPP3
10	NT5E	NT5E	NT5E
11	SLC16A9	SLC16A9	SLC16A9
12	SLCO2A1	SLCO2A1	SLCO2A1
13	LCN6	LCN6	LCN6
14	FGF7	LINC00670	
15	FLJ30064	LRRC17	
16	GNMT	MME	
17	GPAT3	ABCG2	
18	LIPH	ADAMTS15	
19	NQO1	ARRDC3	
20	SLC7A8	C1orf105	
21	TNNT1	MYH6	
22	CPNE5	NAP1L3	
23	BEX1	NPTX2	
24	CCL5	PHLDA1	
25	DIO2	PI16	
26	DPT	SCUBE2	
27		FAM46B	
28		FCGBP	

SVM-RFE: support vector machine-recursive feature elimination; LASSO: least absolute shrinkage and selection operator.

## Data Availability

All the data generated or analyzed during this study are included in this published article.

## Supplementary Materials

Supplementary Materials Additional file 1. ROC curves of the 13 hub genes in the GSE57338 dataset. Additional file 2. The differential expression of 13 hub genes in the GSE57338 dataset. Additional file 3. The detailed process of WGCNA analysis.
