# Supplementary material for: Identification of Biomarkers Associated with Heart Failure Caused by Idiopathic Dilated Cardiomyopathy Using WGCNA and Machine Learning Algorithms
Source: Int J Genomics. 2023 Apr 25;2023:2250772. doi: 10.1155/2023/2250772 (PMC10154102; doi:10.1155/2023/2250772)
Supplement: Supplementary Materials — Additional file 1. ROC curves of the 13 hub genes in the GSE57338 dataset. Additional file 2. The differential expression of 13 hub genes in the GSE57338 dataset. Additional file 3. The detailed process of WGCNA analysis. [file 2250772.f1.docx]

**Additional files**

**Additional file 1.** ROC curves of the 13 hub genes in the GSE57338 dataset.

**Additional file 2.** The differential expression of 13 hub genes in the GSE57338 dataset.

**Additional file 3.** The detailed process of WGCNA analysis.


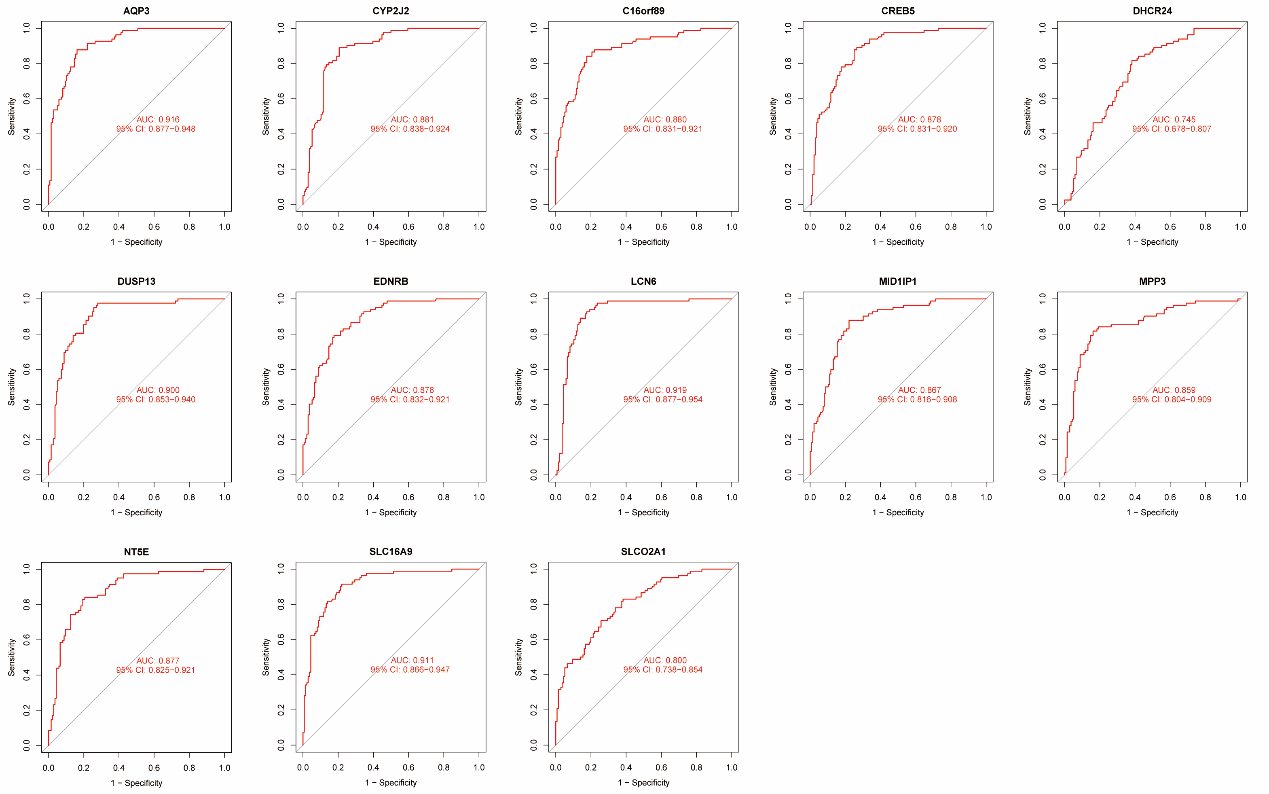


**Additional file 1.** ROC curves of the 13 hub genes in the GSE57338 dataset.


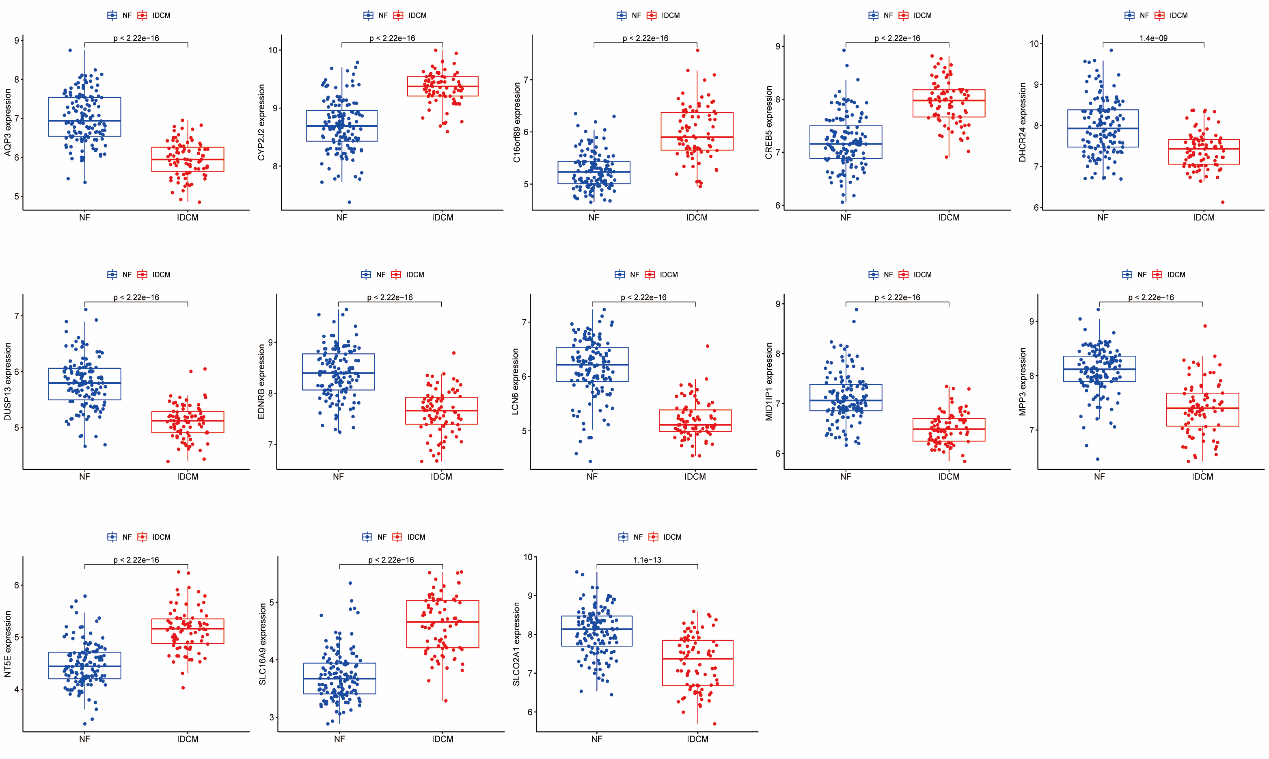


**Additional file 2.** The differential expression of 13 hub genes in the GSE57338 dataset.

**Additional file 3.** The detailed process of WGCNA analysis.

1. **Data input and cleaning**
2.
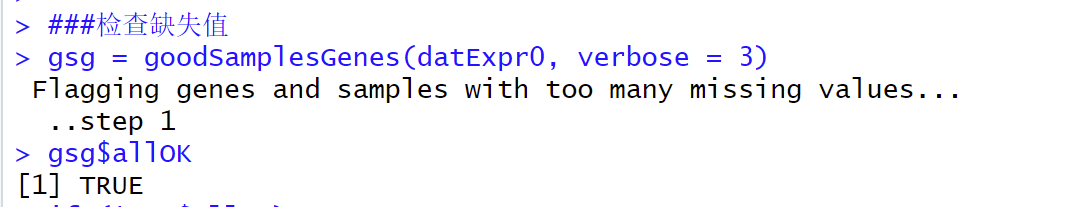
We loaded expression data, then did rudimentary data cleaning and outlier removal. The result of “gsg$allOK” was TRUE. It proved that there was no missing value.
3. We clustered all samples and observed whether there were outliers. Because outlier samples may be those of significant gene expression difference between normal people and patients, we set a large cutting line and did not segment the outlier samples.


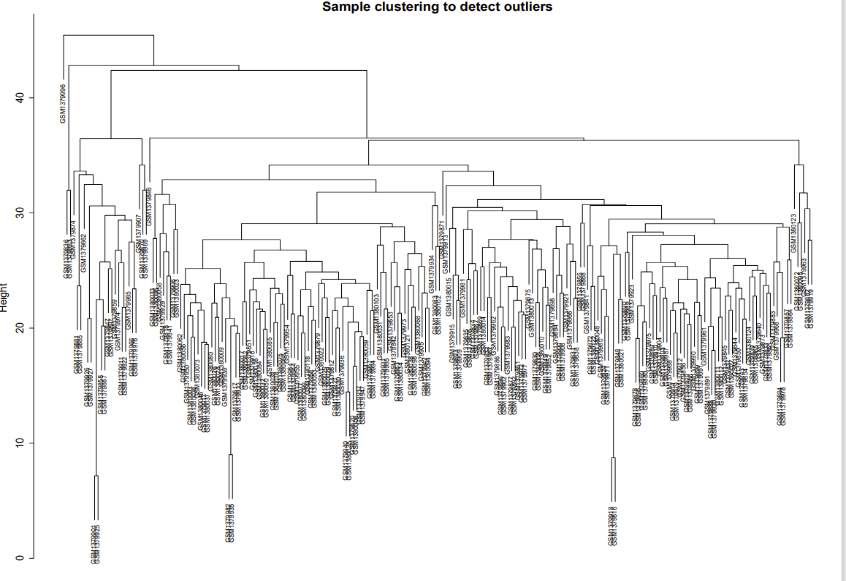


1. We matched clinical characterization data and expression data to reconstruct the sample cluster tree.


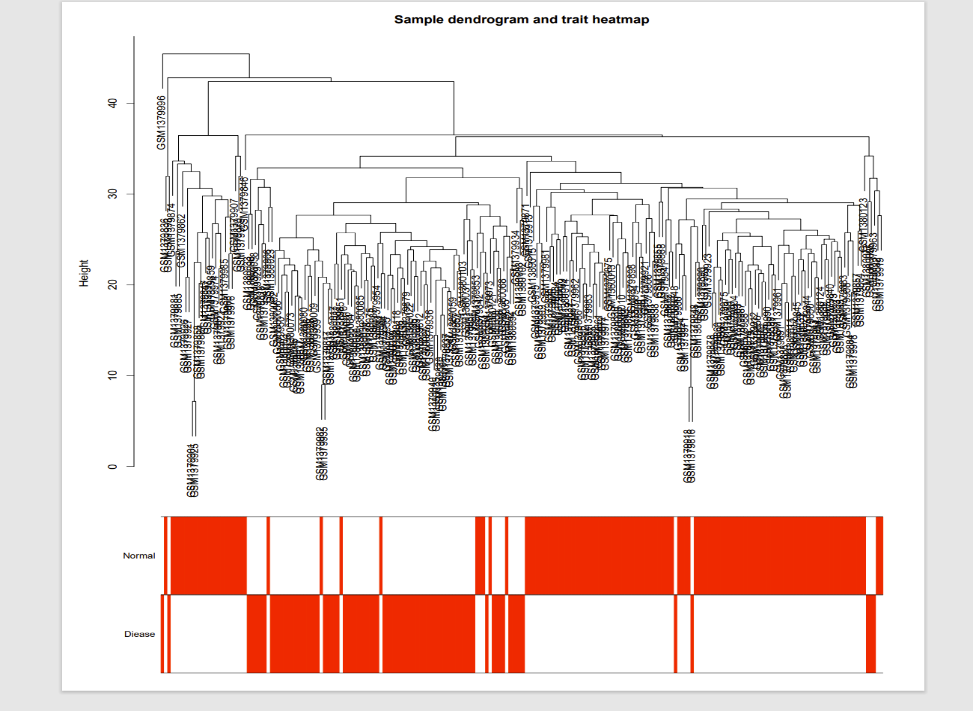


1. **Network construction and module detection**
2. We performed “pickSoftThreshold” in WGCNA to compute β values (range: 1-20) and selected 11 as the optimal soft threshold.


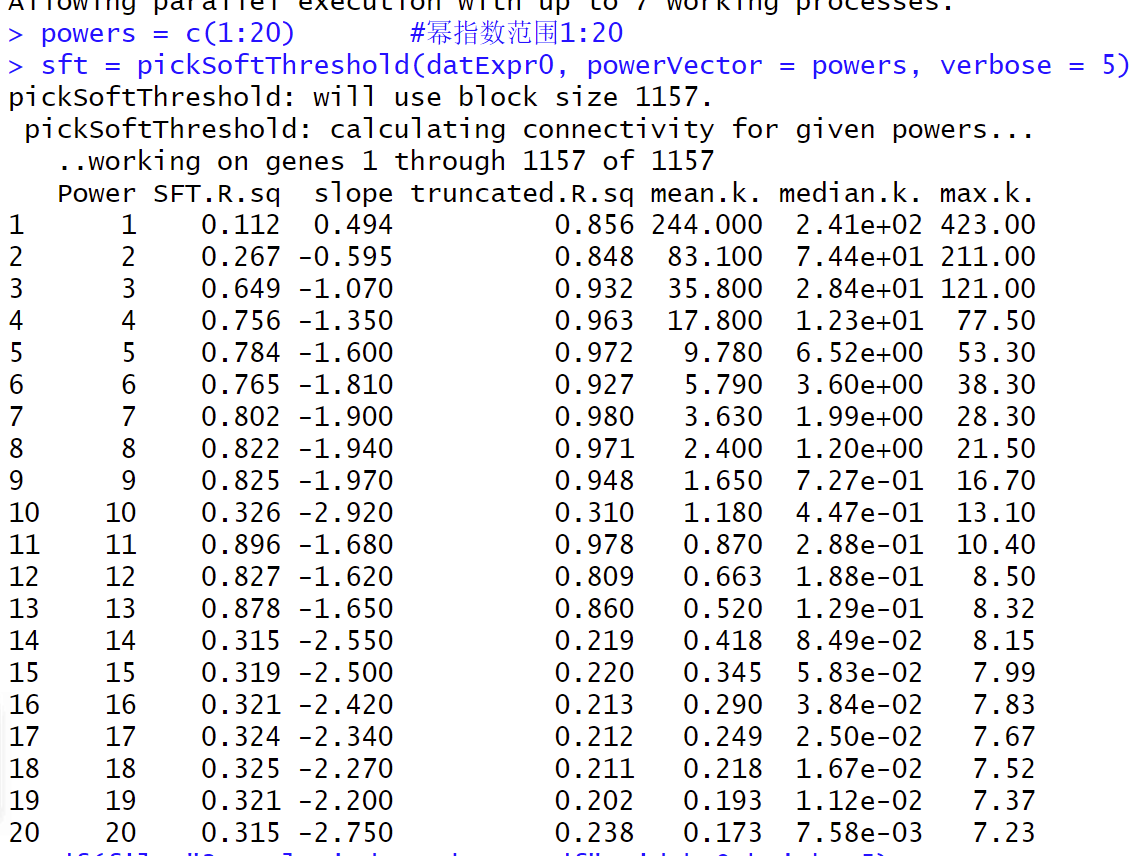


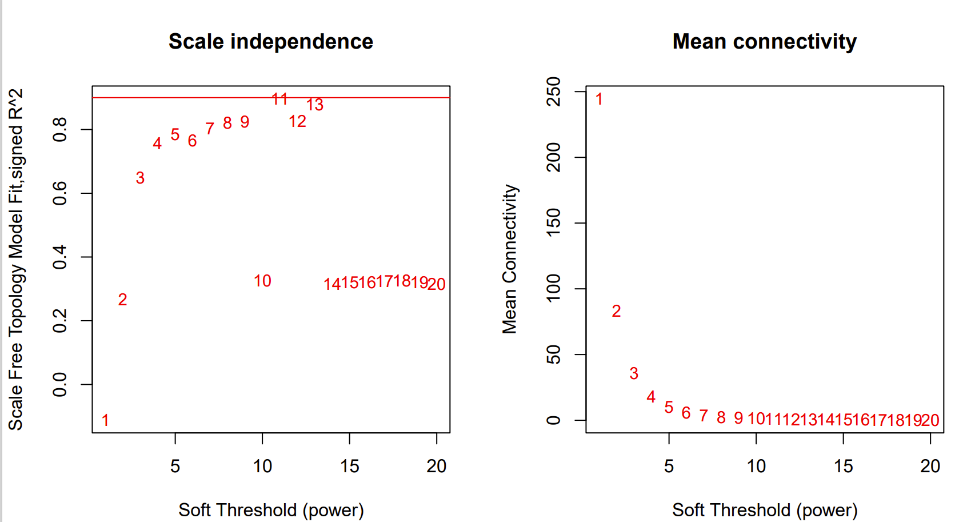


1. To minimize effects of noise and spurious associations, we transformed the adjacency into the Topological Overlap Matrix, and calculated the corresponding dissimilarity.


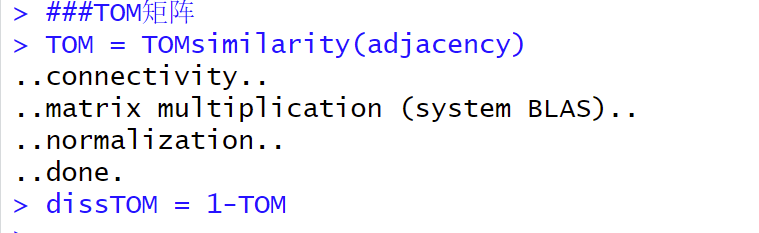


1. We used hierarchical clustering to produce a hierarchical clustering tree (dendrogram) of genes.


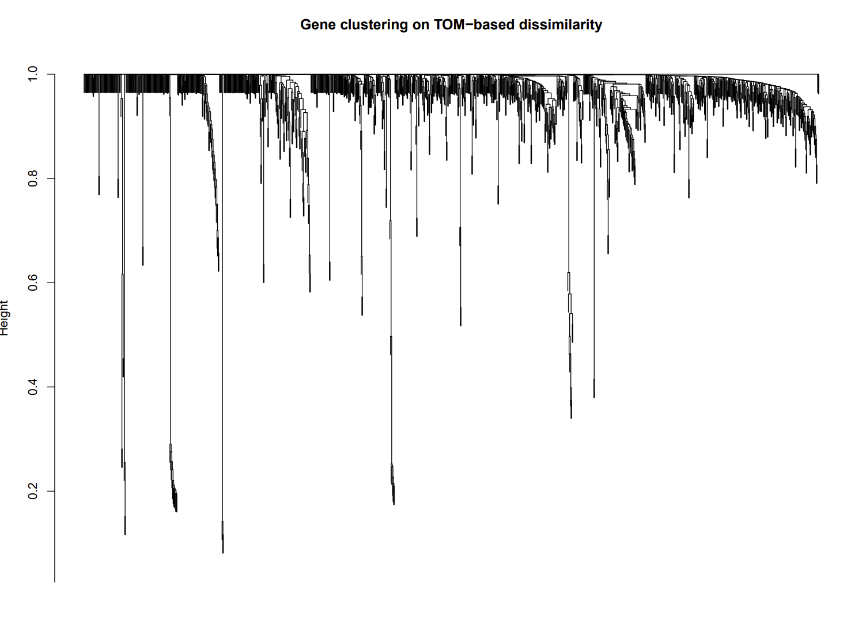


1. We plotted the module assignment under the gene dendrogram.


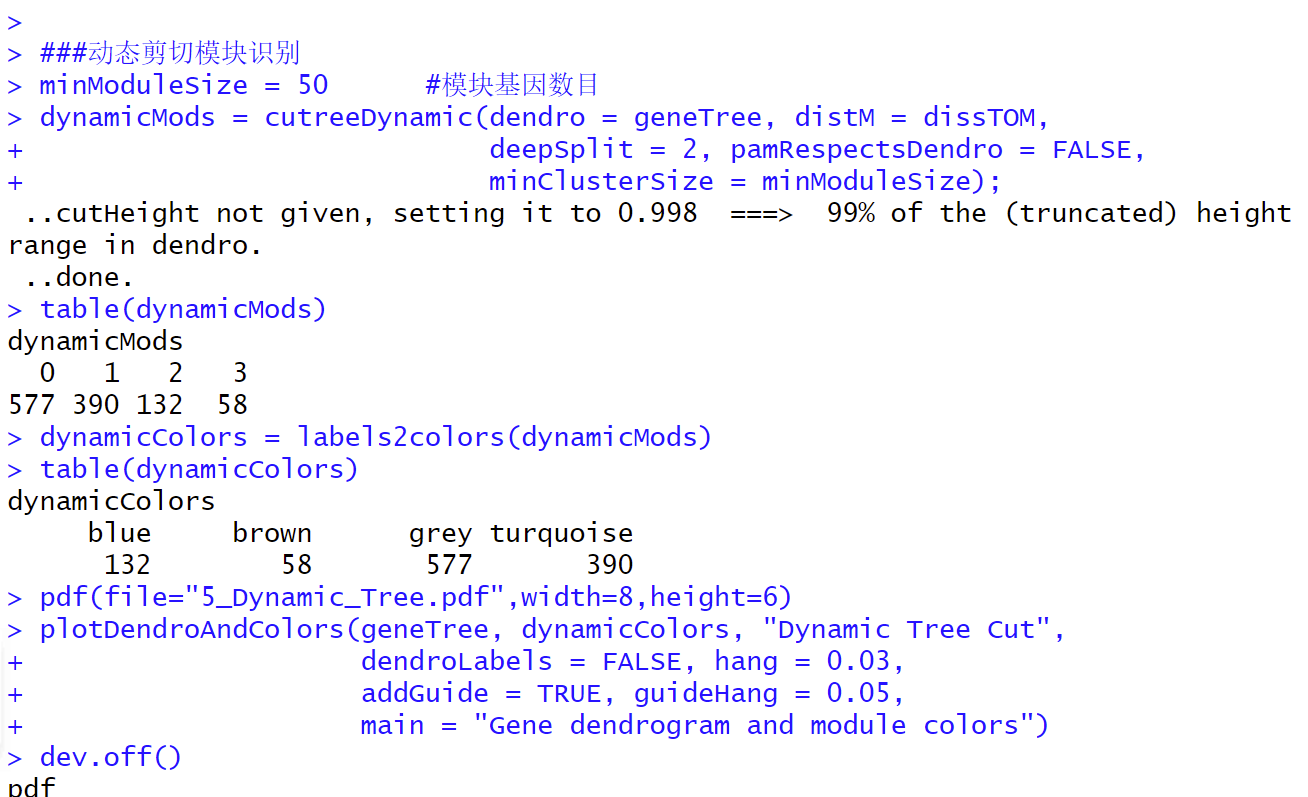


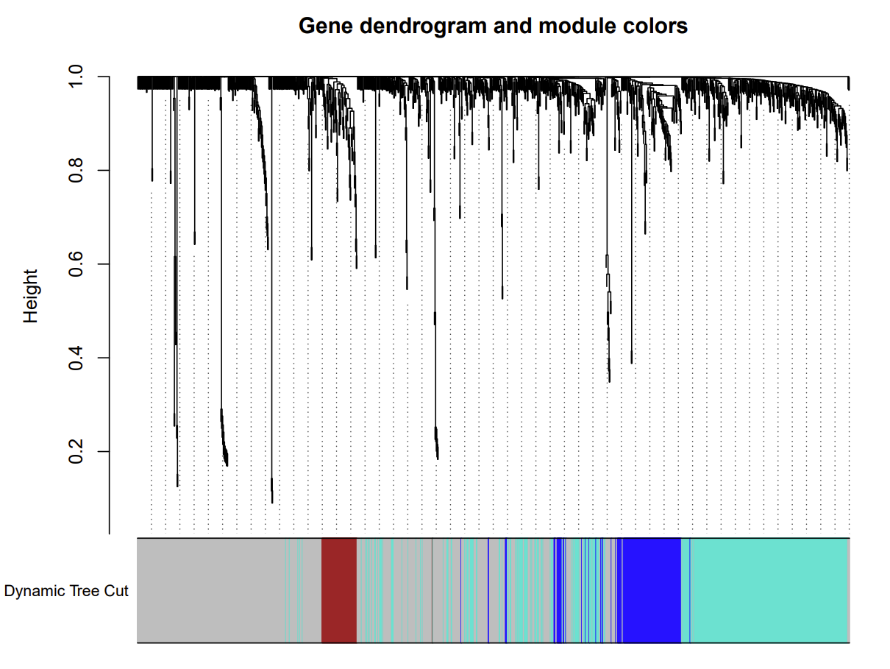


1. We merged modules whose expression profiles were very similar.


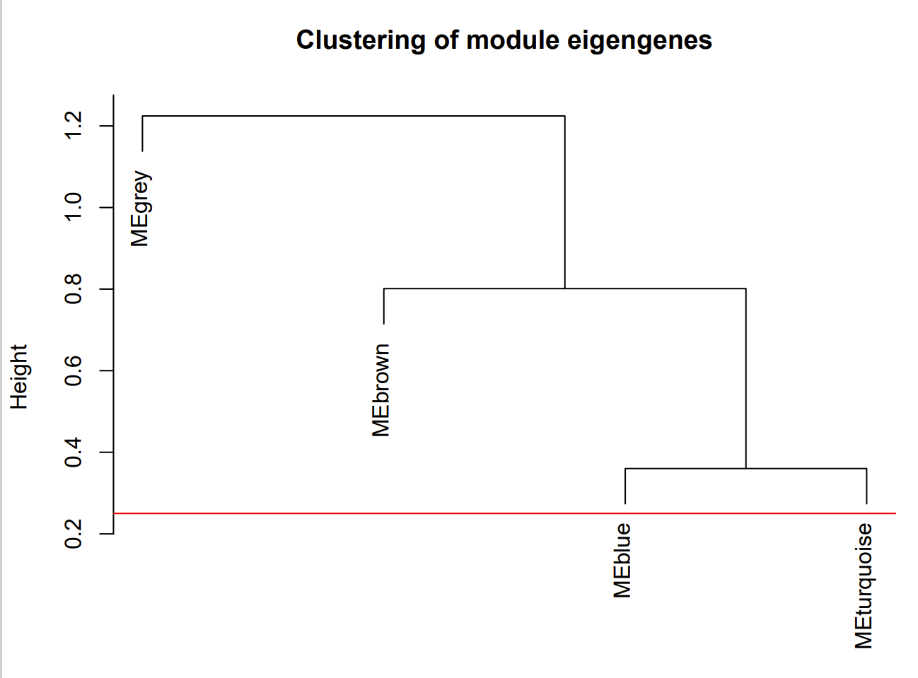


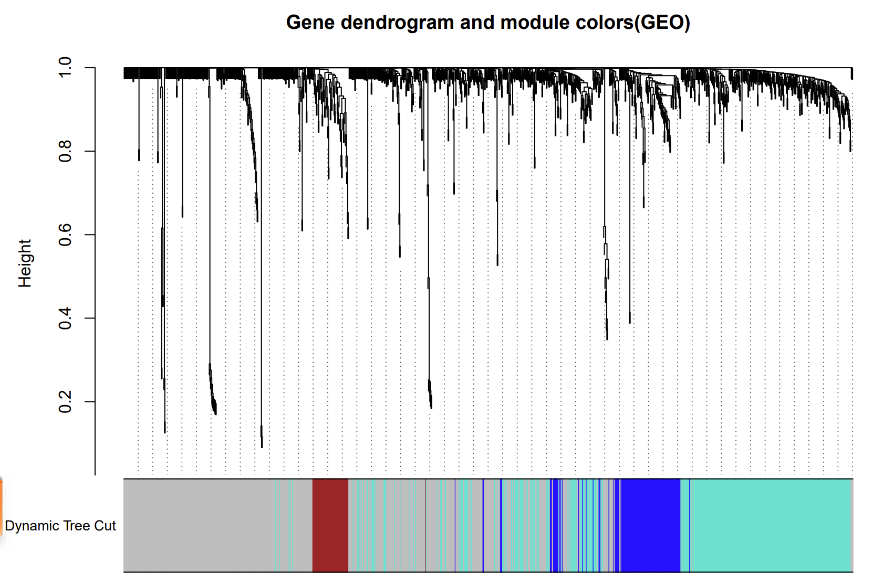


1. **Relating modules to external clinical traits and identifying important genes**
2. After merging similar modules, a total of 4 modules were identified. Based on the module-trait associations, the “MEgrey” module was picked as key module.


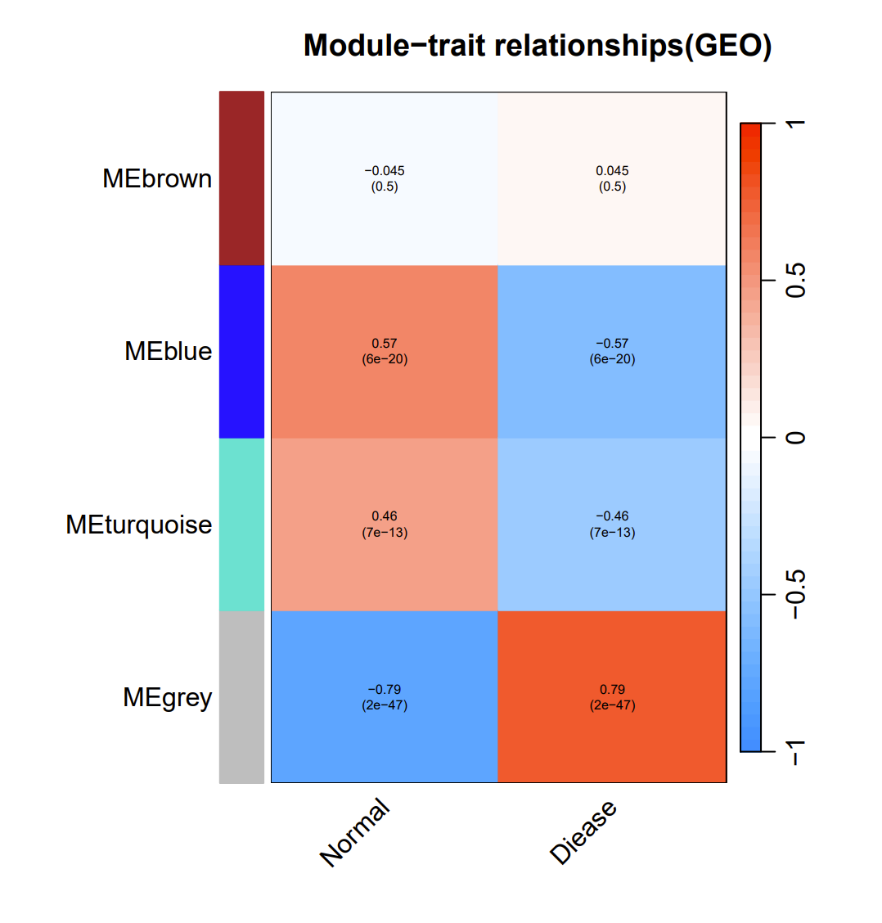


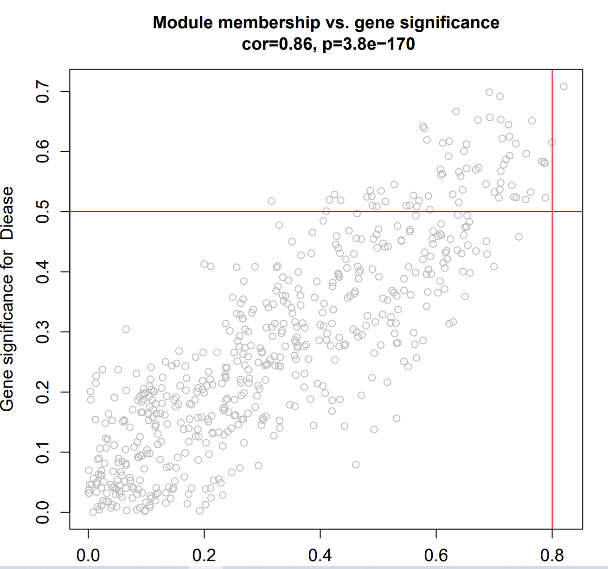

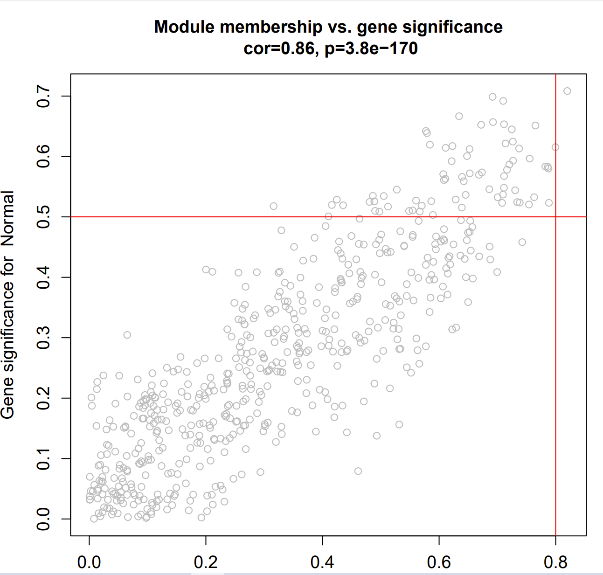


1. Gens in “MEgrey” module.
